# A comprehensive overview on the surgical management of secondary lymphedema of the upper and lower extremities related to prior oncologic therapies

**DOI:** 10.1186/s12885-017-3444-9

**Published:** 2017-07-05

**Authors:** Ramon Garza, Roman Skoracki, Karen Hock, Stephen P. Povoski

**Affiliations:** 1 PRMA Plastic Surgery, San Antonio, TX 78240 USA; 2 Department of Plastic Surgery, Arthur G. James Cancer Hospital and Richard J. Solove Research Institute and Comprehensive Cancer Center, The Ohio State University Wexner Medical Center, Columbus, OH 43210 USA; 3 Division of Oncology Rehabilitation Services, Arthur G. James Cancer Hospital and Richard J. Solove Research Institute and Comprehensive Cancer Center, The Ohio State University Wexner Medical Center, Columbus, OH 43210 USA; 4 Division of Surgical Oncology, Department of Surgery, Arthur G. James Cancer Hospital and Richard J. Solove Research Institute and Comprehensive Cancer Center, The Ohio State University Wexner Medical Center, Columbus, OH 43210 USA

**Keywords:** Lymphedema, Vascularized lymph node transfer, Lymphaticovenular bypass, Lymphogram, complete decongestive therapy

## Abstract

Secondary lymphedema of the upper and lower extremities related to prior oncologic therapies, including cancer surgeries, radiation therapy, and chemotherapy, is a major cause of long-term morbidity in cancer patients. For the upper extremities, it is most commonly associated with prior oncologic therapies for breast cancer, while for the lower extremities, it is most commonly associated with oncologic therapies for gynecologic cancers, urologic cancers, melanoma, and lymphoma. Both non-surgical and surgical management strategies have been developed and utilized, with the primary goal of all management strategies being volume reduction of the affected extremity, improvement in patient symptomology, and the reduction/elimination of resultant extremity-related morbidities, including recurrent infections. Surgical management strategies include: (i) ablative surgical methods (i.e., Charles procedure, suction-assisted lipectomy/liposuction) and (ii) physiologic surgical methods (i.e., lymphaticolymphatic bypass, lymphaticovenular anastomosis, vascularized lymph node transfer, vascularized omental flap transfer). While these surgical management strategies can result in dramatic improvement in extremity-related symptomology and improve quality of life for these cancer patients, many formidable challenges remain for successful management of secondary lymphedema. It is hopeful that ongoing clinical research efforts will ultimately lead to more complete and sustainable treatment strategies and perhaps a cure for secondary lymphedema and its devastating resultant morbidities.

## Background

**Fig. 1:**
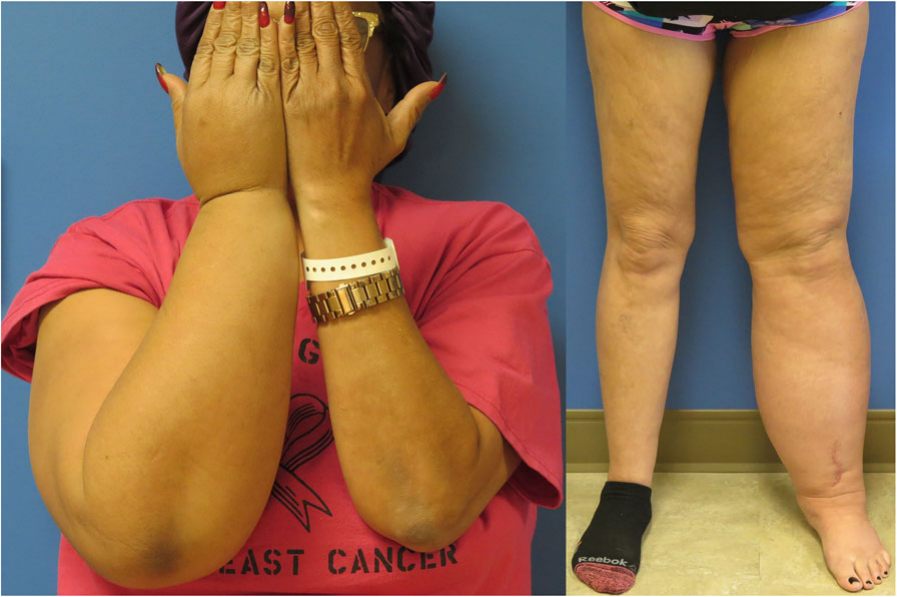
Stage II breast cancer related lymphedema of the right upper extremity (*left*), uterine cancer related lymphedema of the left lower extremities (*right*)

Lymphedema is the buildup of protein rich extracellular fluid within the interstitial compartment of tissues that arises from an imbalance of lymph production and lymph transport to the systemic circulation. Fluid moves via ultrafiltration out of the capillary circulation into the interstitium, then to the lymphatic system, and finally into the systemic circulation [1, 2]. Edema develops when filtration rate exceeds the lymphatic system’s ability to manage fluid balance. Changes in the interstitium subsequently take place that ultimately progress to swelling of the extremity, adipocyte enlargement and irreversible fibrosis. This process can create a cycle of fibrosis leading to further lymphatic transport disruption and worsening lymphedema. Lymphedema is reported to affect 90 million people worldwide. The majority of these cases are secondary lymphedema caused by filiarial disease, a parasitic roundworm infection of the lymphatic system caused by Wuchereria bancrofti. However, in the western world, the majority of cases of extremity lymphedema occur after prior cancer-related surgeries and other adjuvant therapies. Most frequently, these include breast cancer and gynecologic malignancies, as well as melanoma, sarcoma, lymphoma, prostate cancer, urologic cancers, and head and neck malignancies (Fig. 1) [3]. This untoward complication has substantial morbidity for patients and significantly impacts quality of life of those afflicted [1]. It also translates into a significant financial burden for the treatment and maintenance therapy of patients afflicted with this life-long condition. In a study by Shih et al., treatment costs for patients with lymphedema were twice as much as non-lymphedema patients over the course of two years - $23,167 vs $14,877, respectively. It can be assumed that patients will continue to incur costs related to their lymphedema treatment over the course of their lifetime and that the total costs associated with these treatments would be significant. In addition to the monetary impact that lymphedema patients face, there are also significant psychosocial effects associated with lymphedema. Patients with lymphedema are reported to feel depressed, angry, and frustrated. They also commonly have complaints of perceived diminished sexuality as well as social isolation. Some investigators are evaluating how treatments effect the psychosocial well-being of lymphedema patients validating the importance of psychosocial health in this patient population [4, 5].

Currently, there is no cure for lymphedema. However, new surgical treatment options are showing promising results. This comprehensive review will provide general information about secondary lymphedema related to oncologic therapies and discuss treatment options with a focus on surgical treatment.

## The lymphatic system

Until the mid nineteenth century, the lymphatic system was poorly understood. Modern technology and imaging has allowed us to garner a greater understanding of the lymphatic system in structure and function. The lymphatic system is developed embryologically along with the vascular system. Lymphatic vessels parallel the venous system in the extremities. The superficial lymphatic vessels (i.e., primary lymphatics) lack a smooth muscular layer in the vessel wall and thus are dependent on osmotic gradients and hydrostatic pressure differences to aid in movement and absorption of interstitial fluid and proteins. Secondary lymphatic vessels are larger and their walls do have a muscular component (although much thinner than that found in arteries and veins) that aids in propelling fluid in an afferent direction. Additionally, the secondary lymphatics have valves that assist in afferent fluid movement. Primary lymphatics lack valves. Simplistically, the primary lymphatics may be considered the French drains of the body’s immune system, collecting fluid, protein, cells and debris for transport in the secondary lymphatics, which propel fluid unidirectionally to lymph nodes and eventual clearance into the blood stream. The lymphatic system has three primary functions: maintaining fluid balance, serving as a nutritional adjunct, and aiding host defenses against disease [6, 7].

Lymph vessels are ubiquitous in the body with the exception of skeletal muscle. These vessels aid in returning capillary ultrafiltrate and escaped plasma proteins from tissues back into the systemic circulation. This process is important in maintaining balance between interstitial fluid and intravascular volume. Lymphatic vessels are uniquely designed to do this because of fenestrations in their endothelium which allows diffusion of interstitial fluid and proteins into the lumen of the lymph vessel [8]. Lymph fluid is devoid of red blood cells and platelets. Ultimately, these fluids will be returned to the systemic circulation via the connection between the thoracic duct and the left subclavian vein as well as lymphatic connections to the right subclavian vein. There is also some evidence that lymph fluid may enter the venous circulation at the level of the lymph nodes [9].

## Primary lymphedema

**Fig. 2:**
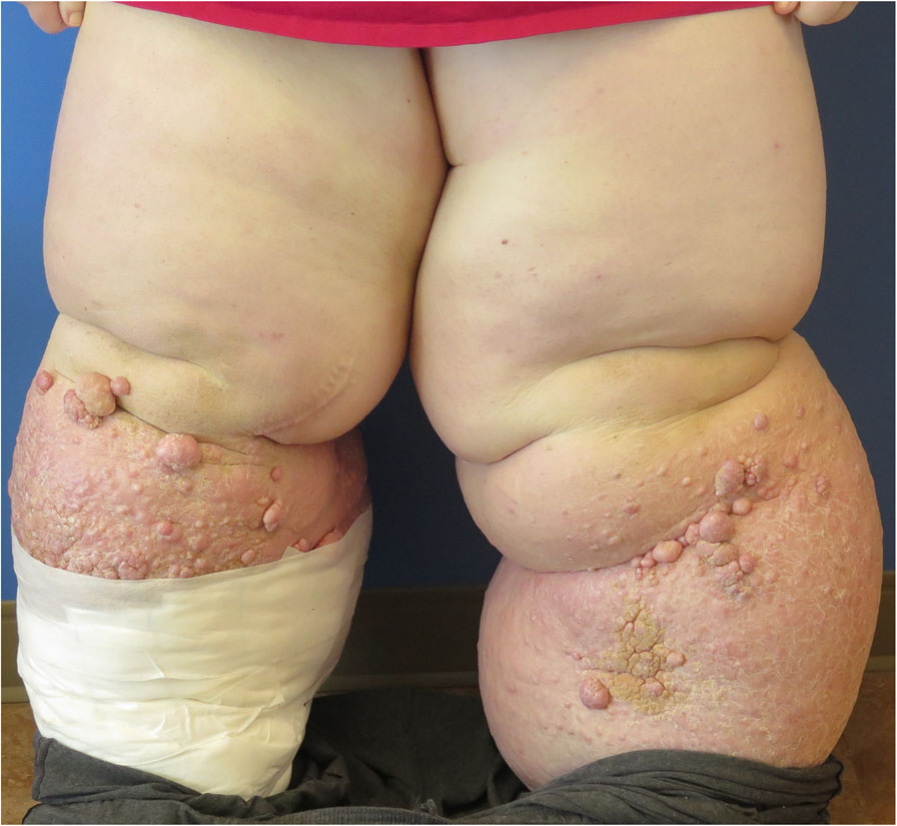
Stage III bilateral lower extremity lymphedema in a 32 year-old patient who was diagnosed in childhood

Primary lymphedema occurs when there is an intrinsic disruption in the lymphatic drainage system. This can be caused by an abnormality in the lymphatic vascular structure or function. The most common classification of primary lymphedema is based on timing of presentation. Congenital lymphedema occurs at birth or shortly thereafter. Lymphedema praecox occurs after birth, but before age 35 (Fig. 2). After age 35, primary lymphedema is defined as lymphedema tarda. Recent studies have identified several genes associated with these syndromes including FLT4 and FOXC2. These associated genes have provided a better understanding of lymphatic system development and structure. These genes may also help with classifying the variations of primary lymphedema [6, 7].

## Secondary lymphedema

Secondary lymphedema is the result of an extrinsic disruption in lymphatic transport. This is usually the result of some form of trauma to the lymphatic system (i.e., surgery, radiation therapy, chemotherapy, or inflammation/scarring from metastases or filarial diseases). In the western world, the most common forms of trauma to the lymphatic system are related to prior oncologic therapies, including cancer surgeries, radiation therapy, and chemotherapy [2, 10]. Worldwide, filarial diseases, which cause inflammation and scarring in the lymphatic system, account for a significant portion of secondary lymphedema patients. The most common organism encountered in this setting is the human parasitic roundworm, Wuchereria bancrofti [2, 10]. If not treated in a timely manner, infection with this parasite leads to elephantiasis. Elephantiasis is the severe debilitating end stage of secondary lymphedema affecting the extremities which resemble the legs of an elephant in shape and associated skin changes.

Secondary lymphedema related to prior oncologic therapies, including cancer surgeries, radiation therapy, and chemotherapy, can have a variable time to onset. In breast cancer patients, it has been reported to occur from as early as 30 days after surgery to as long as 30 years after surgery [11]. However, the majority of patients will experience onset within the first 2–3 years after treatment [12, 13]. Breast cancer related lymphedema typically affects the ipsilateral upper extremity. Breast cancer surgery is also the most common cause of secondary lymphedema in the upper extremity in the western world. Patients who undergo axillary lymph node dissection are at particularly high risk for developing upper extremity lymphedema [14]. Less invasive axillary lymph node surgery, such as a sentinel lymph node biopsy procedure, significantly reduces, but does not eliminate the risk of lymphedema. Sentinel lymph node biopsy does have a lower risk (5–10%) of developing lymphedema compared to complete axillary lymph node dissection (20–50%). In a study by Kim et al., researchers looked at risk factors for the development of lymphedema in breast cancer patients. Three risk factors were found to be statistically significant. They found that patients who had 10 or more lymph nodes removed during axillary lymph node biopsy procedures had an increased risk of developing lymphedema. Two other statistically significant risk factors for the development of lymphedema were adjuvant chemotherapy and supraclavicular radiation therapy. Their study followed patients prospectively for at least 3 years after breast cancer treatment and found that those who had zero or one of these risk factors only had a 3% chance of developing lymphedema. Those with 3 or more of the aforementioned risk factors had a 38% 5-year probability of developing lymphedema [13]. Interestingly, one significant risk factor, absent from the cohort studied, was an elevated body mass index (BMI), due to the overall slender patient population examined in the study. Patients with a BMI higher than 30 kg/m^2^ are up to 3.6 times more likely to develop lymphedema. Moreover, patients who are obese at the time of breast cancer diagnosis are at greater risk of developing secondary lymphedema than patients who gained weight to an obese level after breast cancer diagnosis. Losing weight after the diagnosis of breast cancer did not change this risk [11, 15, 16].

Lower extremity lymphedema most commonly occurs from the treatment of gynecologic cancers, urologic cancers, melanoma, and lymphoma. Ingiuinal, pelvic, and periaortic lymph node dissections put patients at particularly high risk for developing lymphedema in the lower extremities. Similar to upper extremity lymphedema, combinations of node dissection, local radiation therapy and chemotherapy are also risk factors for lymphedema in the lower extremity. The typical time to development of lymphedema is slightly quicker with 80% of patients presenting within the first 12 months after treatment [16].

## Diagnosing lymphedema

The ubiquitous characteristic of lymphedema is swelling. Because of this, lymphedema is clinically diagnosed by determining that a limb is in fact swollen, and eliminating other etiologies. Limb circumference differences of 2 cm, limb volume differences of 200 mL, or a 5% volume change are some of the objective ways that clinicians use to diagnose lymphedema [17–20]. However, there are inconsistencies in the literature and in practice regarding using these diagnostic guideline. This is further complicated as other conditions can cause limb swelling. In a study by Maclellan et al., only 75% of patients diagnosed with and referred to a lymphedema specialist truly had lymphedema. The other etiologies of limb swelling that were incorrectly diagnosed as lymphedema included: venous stasis, lipedema, obesity, injury, rheumatologic disease, and vascular malformations [21]. Poorly controlled congestive heart failure will also increase the ultrafiltration rate in the capillary beds leading to excess swelling and acutely increased protein content in lymph fluid.

In addition to volume changes, patient symptoms also include: sensations of heaviness, achiness, decreased range of motion, skin changes, and recurrent cellulitis.

The pathophysiology of edema and lymphedema is important to understand when approaching patients with swelling of an extremity related to excess fluid accumulation in the tissues. Rockson et al. explained that all edema in the extremities is created from a relatively incompetent lymphatic system that is overwhelmed by fluid microfiltration in the tissues. This can either be from an inadequate lymphatic system that cannot bear the load of a normal amount of fluid in the extremities (lymphedema) or from a normal lymphatic system that is overwhelmed with a high microcirculation fluid accumulation from a myriad of possible conditions, such as heart failure, kidney failure, liver disease, and malnutrition to name a few [6]. Because of the numerous etiologies of edema and swelling, particularly in the lower extremities, true lymphedema can be misdiagnosed or other conditions that are not lymphedema are wrongly classified as lymphedema. When evaluating a patient for lymphedema, clinicians should first rule out other conditions that similarly present with swelling of the affected extremities. Clinicians who care for and see lymphedema patients should have in place a protocol for evaluating these potential confounding conditions. This should also include an assessment of the venous system of the affected extremity.

**Fig. 3:**
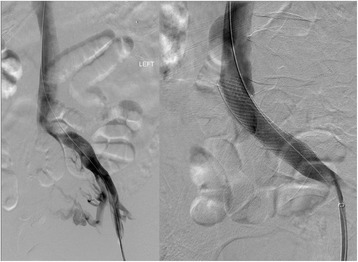
Angiogram of patient with May-Thurner Syndrome, demonstrating a narrowed left iliac vein before (*left*) and after angioplasty and stent placement (*right*)

Venous insufficiency, in the lower extremity can mimic lymphedema in its fluid accumulation, in spite of a functional lymphatic system. Relative venous outflow obstruction, such as May-Thurner Syndrome in the lower extremity has been shown to result in a swollen lymphedematous limbs, in the absence of any injury to the lymphatic system [22]. Addressing the underlying venous problem is the treatment of choice, such as angioplasty and stenting of the relative outflow obstruction (Fig. 3). Similar procedures in the obstructed venous systems of the upper extremity are not well studied and most of the published literature is in relation to thrombosed hemodialysis arterio-venous fistulas [22, 23]. Recently researchers in Brussels have identified a subgroup of breast cancer patients who display symptoms of relative venous outflow obstruction resulting in upper extremity lymphedema. Axillary scar release and the addition of soft tissue to minimize recurrent scar formation has been successful in alleviating the upper extremity swelling without any interventions directed at the lymphatic system in this subset of patients. By correctly diagnosing these patients, unwarranted lymphedema treatment procedures and testing can be avoided [24].

## Monitoring lymphedema

One of the biggest challenges in managing and monitoring lymphedema is volume measurement. Currently, there is no gold standard for diagnosing and monitoring the progression of lymphedema. There are numerous methods used to both diagnose and monitor lymphedema [25]. The most common methods include arm circumference measurements, water displacement, tissue tonometry, perometer, bioimpedance spectroscopy, contrast enhanced magnetic resonance lymphangiography, and indocyanine green lymphangiography. Again, centers focusing on lymphedema treatment should have standardized methods of evaluating and monitoring these patients.

**Fig. 4:**
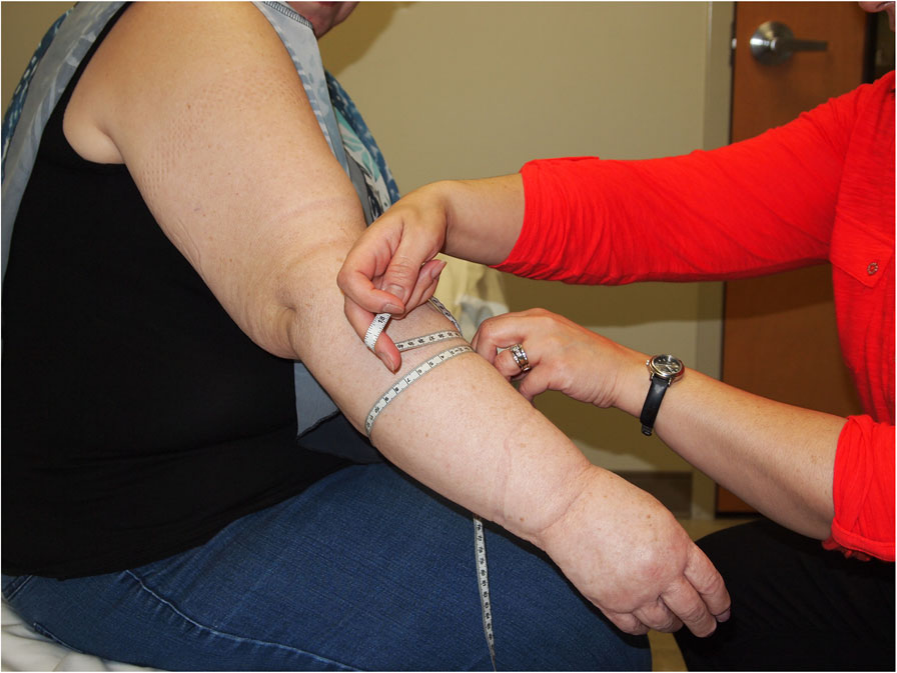
Certified lymphedema therapist performing circumference tape measurements on a patient with breast cancer related right upper extremity lymphedema

Circumference measurement is the most frequently used technique due to the low expense and ease of use. Circumferential measurements are either taken at boney landmarks or established locations along the limb (Fig. 4) [26]. This method requires operator experience and can be time consuming. There is also potential variability in the location that a measurement is taken along the extremity and the relative tension placed on the tape measure may affect the accuracy. It is ideal to perform as many measurements as is practically possible (in 4–9 cm intervals along the length of the limb), as a greater number of measurements will allow greater accuracy when using the measurements to calculate the limb volume using a modified cone equation.

Water displacement involves submerging the affected limb in a container with a preset volume of water. The amount of water displaced, represents the total volume of the portion of the limb that is submerged. One of the challenges with this technique is inter-rater and intra-rater reliability as it may be difficult to identify a reliable landmark along the limb that can serve as a reproducible point to which the limb is submerged during each subsequent measurement. Water displacement is also limited in its use because it is impractical in the clinical setting. The device is bulky, messy and is contraindicated for individuals with open wounds due to the need to disinfect between patients. Although it is considered the “gold standard”, its use is more common in research than clinical practice [27, 28].

Tissue tonometry measures the ability to compress the skin to a specific depth at a given force. As a handheld device, it allows for each operator to apply different amounts of pressure with measurement introducing error. Chen et al. demonstrated poor intra-rater reliability of tissue tonometry (intraclass correlation coefficients of 0.66–0.88), which the authors attributed to operator variation in the application of the device. Tonometry cannot differentiate between excess tissue rigidity due to fluid or fibrosis. As such, various stages of lymphedema may have differing values with the tonometer [29]. This method does not provide any volumetric data for the affected limb.

**Fig. 5:**
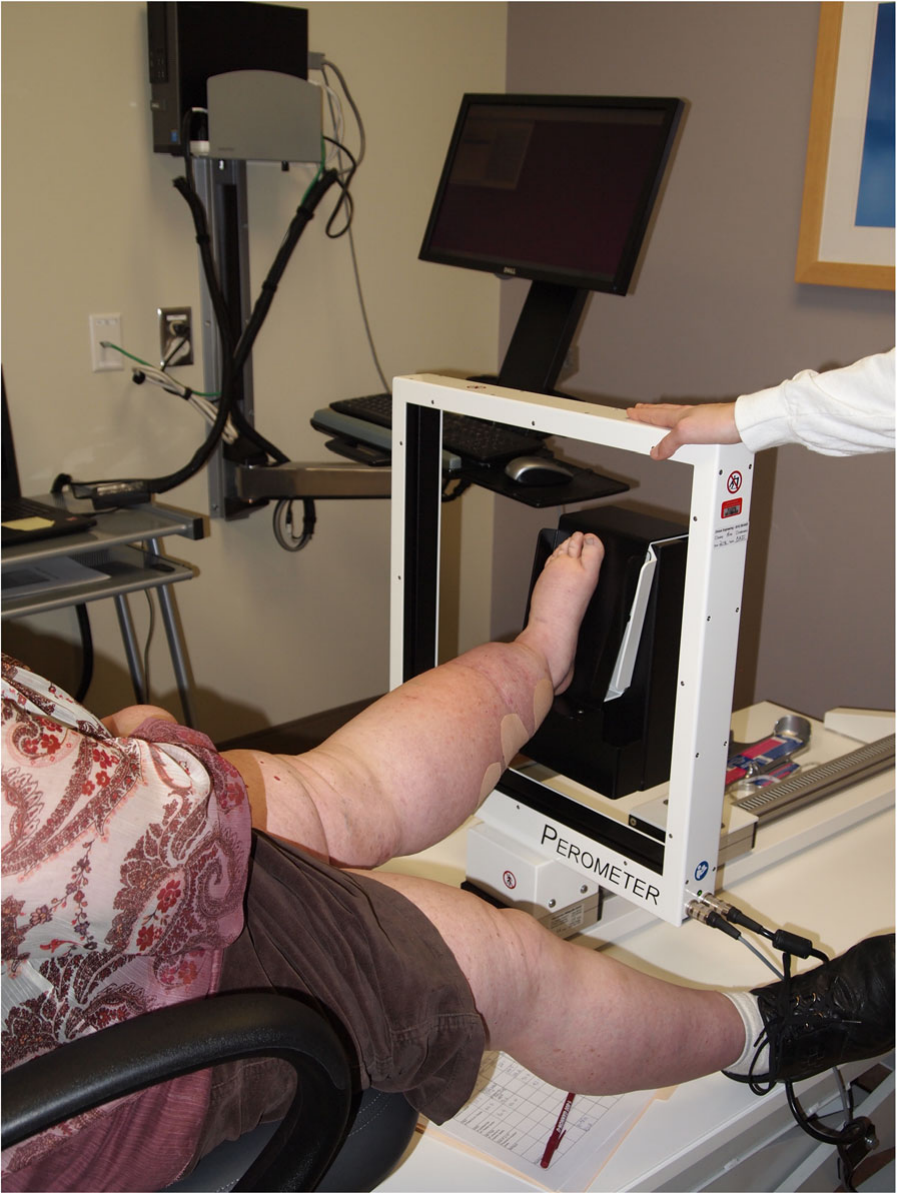
Certified lymphedema therapist performing left lower limb measurement using a perometer

Perometery uses infrared optoelectronic technology to detect changes in limb volume. It uses 360 degrees of infrared light and takes surface measurements at 0.5 cm increments. Volume is then calculated from this information [30, 31]. The measurement is rapid and precise but the machine is bulky and expensive making it difficult for widespread use (Fig. 5). Similar to tape measurements, it is also limited in its inability to distinguish volume changes from weight gain versus edematous changes [30].

Bioimpedance spectroscopy (L-Dex) uses the principle of resistance to electrical current to detect the presence of interstitial fluid over time. As lymphedema worsens and fluid accumulates in the tissue, the resistance to electrical current decreases over time [17]. Ridner et al. demonstrated that patients using bioimpedance to monitor their lymphedema had higher rates of compliance with treatment. Unfortunately, bioimpedance fails to consider the change in tissue composition that accompanies lymphedema progression and fibrotic tissue may falsely increase resistance giving the appearance of response to treatment despite worsening of lymphedema [32]. Furthermore, this method is reported to only have a 66% sensitivity for diagnosing true lymphedema [17]. It also has no utility in diagnosing lymphedema when bilateral limbs are affected, as a control limb is required to use as a reference.

Contrast enhanced magnetic resonance lymphangiography involves an interstitial injection of a contrast agent and T1 weighted MR imaging of contrast uptake into the lymphatic system. Due to the small size of the contrast molecules, uptake into the local vasculature may confound image interpretation [33]. Adding an intravenous dye injection to the examination and utilizing subtraction methods can aid in the differentiation between lymphatic channels and blood vessels. While these imaging studies provide excellent views of lymphatic vessels, the invasive nature, exposure to contrast and expense of the lengthy imaging studies make this method less feasible for tracking lymphatic disease over time.

**Fig. 6:**
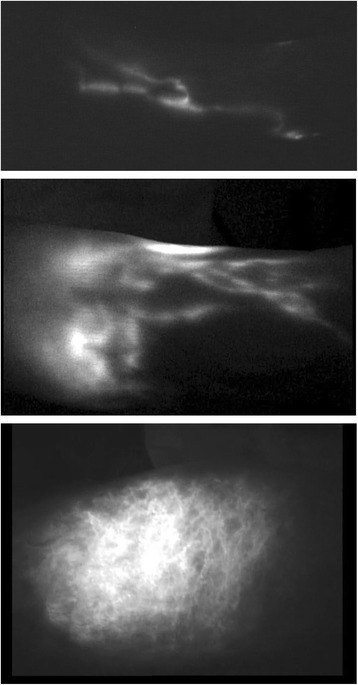
ICG lymphogram (MDACC Staging) Stage I: many patent lymphatic vessels, with minimal, patchy dermal backflow of a left lower extremity (*top*), stage II lymphedema ICG lymphogram showing moderate number of patent lymphatic vessels, with segmental dermal backflow (*center*), stage IV lymphedema ICG lymphogram showing no patent lymphatic vessels, with severe dermal backflow (*bottom*)

Indocyanine green (ICG) lymphangiography involves the injection of a contrast agent into the interstitial fluid and then monitoring flow of protein bound dye in the superficial lymphatic channels below the dermis. This uses near infrared cameras to detect the fluorescence from protein bound, excited ICG molecules. It allows real time visualization of lymphatic flow without exposure to radiation. Surgeons have found this useful in planning surgical options for lymphedema and especially useful for intraoperative planning during various lymphovenous anastomoses procedures [31, 34, 35]. Various dermal backflow patterns have been identified to correlate with the extent of disease progression (Fig. 6) [16, 35, 36]. This tool has shown great promise and accuracy in predicting progression of subclinical lymphedema to clinically significant disease according to the pattern of dermal backflow. Because ICG lymphangiography is limited to visualization of the superficial lymphatic system, with penetration of up to 10 mm, it provides an incomplete picture of the lymphatic system. The invasive nature, variability of clinician interpretation, and expense associated with acquiring the necessary imaging equipment make this method less suitable for screening and long term monitoring of lymphedema patients. This method is being explored also by certified lymphedema therapists to guide manual lymphatic drainage (MLD).

**Fig. 7:**
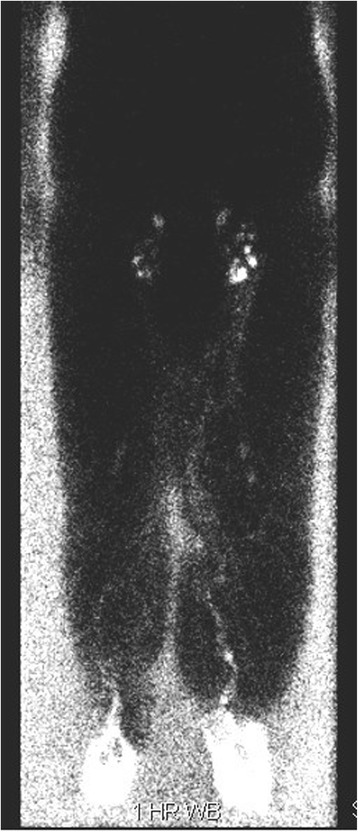
Bilateral lower extremity lymphoscintigram of patient with bilateral congenital lower extremity lymphedema tarda, demonstrating lymphatic webbing, dermal backflow and secondary nodal drainage basins. Note the presence of groin nodes on this 6 h post-injection image

Traditionally, radionuclide lymphoscintigraphy has been widely used for confirming lymphedema in the swollen extremity, demonstrating abnormally slow lymphatic transport. Filtered colloid, Technecium-99 m sulfur colloid, is injected subdermally into the affected, and usually the unaffected control, limb. Lymphoscintigraphy relies on the lymphatic system’s ability to transport large radiolabeled protein or colloid molecules from the interstitial space, through nodal basins, back to the vascular compartment (Fig. 7). The radiolabels can be followed using the gamma camera to detect the radioactivity. Snap shots in time capture the function of the lymphatic system. The existence and function of the lymph node basins can then be analyzed utilizing multiple scans over several hours. Calculation of the transport index is useful to semi-quantitatively determine the severity of lymphedema [37]. Images may be able to provide information about potential anatomic abnormalities such as obstructions, lymphatic dilatation or a reduction in the number of visualized lymphatic channels or lymph nodes. However, the resolution of radionuclide-based imaging is suboptimal and detailed anatomic information, particularly of individual lymphatic channels, can usually not be demonstrated. This technique is invasive, time consuming, and generally does not aid in surgical planning. Still, it can be useful to determine if there are functional lymphatics in the affected extremity. Lymphoscintigraphy may be a useful adjunct for the diagnosis of lymphedema in select patients.

## Lymphedema staging

Lymphedema staging systems are important tools in the management of the disease. Depending on how advanced the disease process is will dictate options available for treatment.

The International Society of Lymphology has established a staging system for lymphedema. This staging system is the most widely utilized staging system for identifying the progression and/or severity of disease, and classifies lymphedema into four clinical stages:

Stage 0: Latent or subclinical condition where swelling is not present despite impaired lymphatic transport. It may exist months or years before overt edema occurs.

Stage I: Early accumulation of fluid relatively high in protein content (i.e., compared to venous edema). Edema subsides with limb elevation. Pitting can be present.

Stage II:

- Early – Pitting is present which does not resolve with elevation alone.
- Late – Tissue fibrosis develops, pitting may or may not be elicited.

Stage III: Lymphostatic elephantiasis where pitting is absent. Trophic skin changes, lipodystrophy, warty skin overgrowths develop. In this system, Stage III is the most severe form of lymphedema and is mostly associated with the filarial cause of secondary lymphedema [28].

Within each stage, severity based on volume excess as compared to the normal may be sub-classified as minimal (<20% volume excess), moderate (20–40% volume excess) or severe (>40%) volume excess.

With the increased use of technology in diagnosing lymphedema and planning its management, new staging systems have been developed. Campisi et al. has described a staging system that uses clinical presentation and lymphoscintigraphic patterns to help classify lymphedema and assist with clinical management [38].

- Stage IA: No clinical edema despite the presence of lymphatic dysfunction as demonstrated on lymphoscintigraphy.
- Stage IB: Mild edema that spontaneously regresses with elevation.
- Stage II: Persistent edema that regresses only partially with elevation.
- Stage III: Persistent, progressive edema; recurrent erysipeloid lymphangitis.
- Stage IV: Fibrotic lymphedema with column limb.
- Stage V: Elephantiasis with severe limb deformation, including scleroindurative pachydermitis and widespread lymphostatic warts.

Campisi further correlated stage of lymphedema with amount of volume excess:

- Stage I: 0–20% volume excess.
- Stage II: 21–40% volume excess.
- Stage III: 41–60% volume excess.
- Stage IV/V: >61% volume excess.

Chang et al. has devised a classification scheme using ICG lymphangiography to assist with surgical planning in lymphedema of the arm (Fig. 6) [31].

- Stage I: Many patent lymphatic vessels, with minimal, patchy dermal backflow.
- Stage II: Moderate number of patent lymphatic vessels, with segmental dermal backflow.
- Stage III: Few patent lymphatic vessels, with extensive dermal backflow involving the entire arm.
- Stage IV: No patent lymphatic vessels seen, with severe dermal backflow involving the entire arm and extending to the dorsum of the hand.

These staging systems are important for both classifying degree of lymphedema, communicating disease severity as well as guiding decision making for clinicians. Advanced stages of lymphedema may respond less well to certain surgical interventions such as lymphovenous anastomosis than earlier stages of lymphedema.

For clinically evident lymphedema, the patient’s primary measure of worsening or improving lymphedema will focus on the change in volume excess. This should also serve as one of the primary longitudinal measures for clinicians when evaluating lymphedema patients both initially and in the long term. This is the amount of volume excess when compared to the contralateral, unaffected, limb or preferably to the same limb, prior to the onset of lymphedema, when available. This is termed Volume Differential (VD). This is calculated as follows:

$$ \frac{\left(\mathrm{Affected}\ \mathrm{limb}\ \mathrm{volume},\hbox{--}, \mathrm{Unaffected}\ \mathrm{limb}\ \mathrm{volume}\right)}{\mathrm{Uanffected}\ \mathrm{limb}\ \mathrm{volume}}\  x100= VD\ \left(\%\right) $$

To evaluate patients postoperatively and determine response to an intervention a Volume Differential Reduction (VDR) is often calculated. This is calculated as follows:

$$ \frac{Preop\  VD- Postop\  VD}{ Preop VD} x\ 100= VDR\ \left(\%\right) $$

These values allow clinicians to follow objective measurements postoperatively and gauge response to treatment [31].

One criticism of the above formulas is that they do not account for a change in volume due to a change in the patient’s BMI over time. Significant changes in BMI, not unusual in lymphedema patients, are important to consider when assessing a patient’s limb volume change over time. This is accounted for when applying the weight-adjusted volume formula (WAC):

$$ \frac{A2 xW1}{A1 W2}-1= WAC $$

- A1 = Preoperative Arm Volume.
- A2 = Postoperative Arm Volume.
- W1 = Preoperative Weight.
- W2 = Postoperative Weight.

The WAC formula is also useful in patients who have had bilateral breast surgery where both limbs could be affected by lymphedema. In these instances, there is no limb that can be used as a control [39].

Additionally, subjective measures are also assessed in follow up exams when managing lymphedema patients. Specifically, symptoms of changes in “heaviness” of the limb, how clothes fit, volume/size of the limb, pain, discomfort, range of motion and dexterity are assessed.

One important measure that is often overlooked clinically is the quality of life of patients with lymphedema. The quality of life measure for limb lymphedema, commonly referred to as the acronym LYMQOL, is useful in evaluating this measure and should be administered regularly. It was developed in the United Kingdom by clinicians who evaluate and treat patients with lymphedema. The tool has questions that the patient answers in the four following domains: 1) symptoms; 2) body image/appearance; 3) function; and 4) mood. This tool can be useful in decision-making regarding intervention and treatment, measuring responsiveness to treatment, and evaluating cost-effectiveness of treatments [40].

## Management strategies

The main goal of treatment of lymphedema is volume reduction of the affected limb and an improvement in patient symptoms as well as a reduction of or elimination of recurrent infections for those patients suffering from these. Certain therapies have been shown to provide patients with improvement in symptoms and clinically relevant volume reductions in their affected extremity. These interventions are categorized either as non-surgical (conservative) therapy or as surgical therapy. The majority of patients with lymphedema are managed non-operatively leaving surgery as a secondary option for those recalcitrant to initial conservative measures or those that plateau at a level unsatisfactory to the patient despite strict adherence to compression and manual lymphatic drainage regimens [21].

### Non-surgical management

#### Medical management

Currently, there is no data to support use of medications in the routine treatment of lymphedema. Several studies evaluated the use of diuretics compared to placebo and found no difference in outcomes and some believe it may actually worsen lymphedema and fibrosis by concentrating protein in the extracellular space [41]. Diuretics have been anecdotally used for early treatment of lymphedema, but use of diuretics in the long term is not recommended. Coumarin may have some benefit in minor volume reduction; however, it has a side effect of hepatotoxicity and for this reason it has been avoided in lymphedema patients. Antibiotics are used in acute cases of cellulitis and there are recommendations for patients to be on prophylactic antibiotics if 3 or more episodes of cellulitis occur over the course of one year, as the incidence of cellulitis has been linked to progression of lymphedema [42–44].

#### Complex decongestive therapy

A comprehensive approach to the management of lymphedema is referred to in the literature by a variety of terms, including complex lymphedema therapy, complete or complex decongestive therapy (CDT), or decongestive lymphatic therapy [45–47]. CDT is divided into two phases. Phase I is the reduction phase while Phase II is the maintenance phase, which includes strategies for long term management. CDT components include manual lymphatic drainage (MLD), compression therapy, exercise and skin care as well as range of motion exercises, breathing and posture exercises, and education [48, 49].

**Fig. 8:**
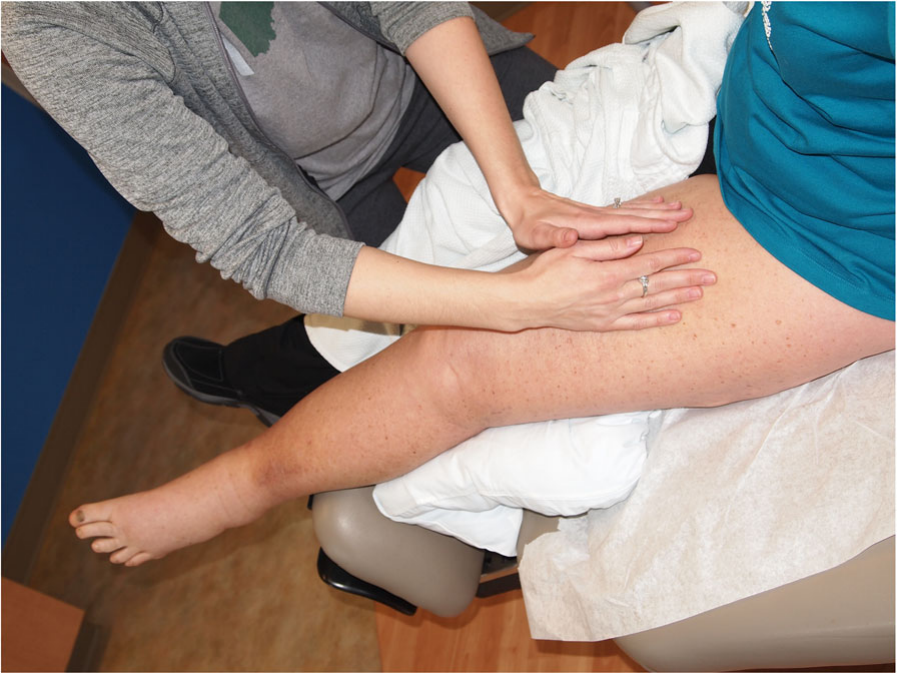
Certified lymphedema therapist performing manual lymphatic drainage on a left lower extremity

MLD involves slow, very light repetitive stroking and circular massage movements done in a specific sequence to clear proximal congestion and redirect fluid to lymphatic beds/pathways with capacity to absorb the extra volume (Fig. 8) [40, 46]. The efficacy and optimization of MLD is now being supported by the use of lymphatic fluid tracing. The injection of ICG has supported MLD as a way to facilitate lymphatic flow as well as demonstrate which lymphatic channels may best optimize MLD treatment [50–52].

**Fig. 9:**
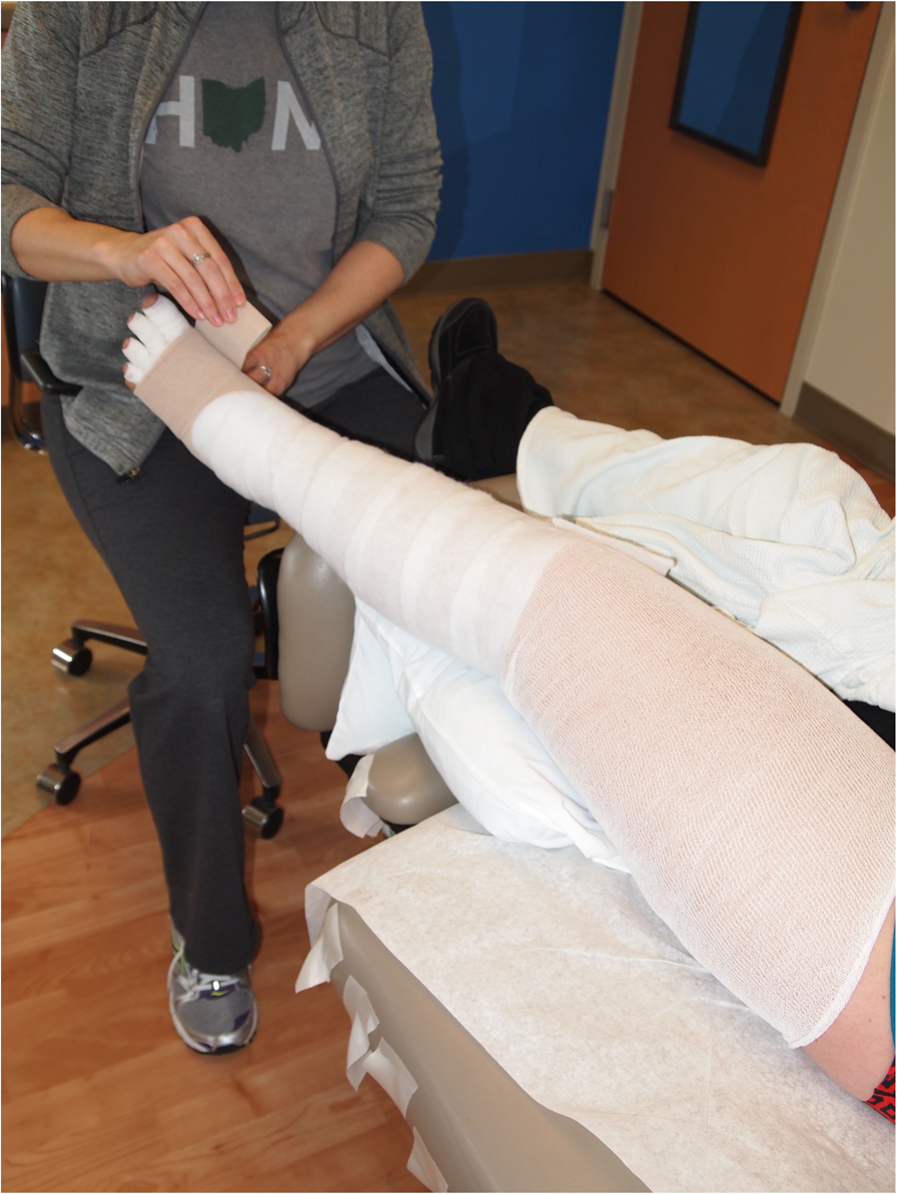
Certified lymphedema therapist applying short stretch compression bandages to a patient’s left lower extremity with cervical cancer related lymphedema

Compression therapy is used in conjunction with MLD. During Phase I, only low-stretch bandages are utilized, which provide a low resting pressure on a limb but a high working pressure (Fig. 9) [53, 54].

**Fig. 10:**
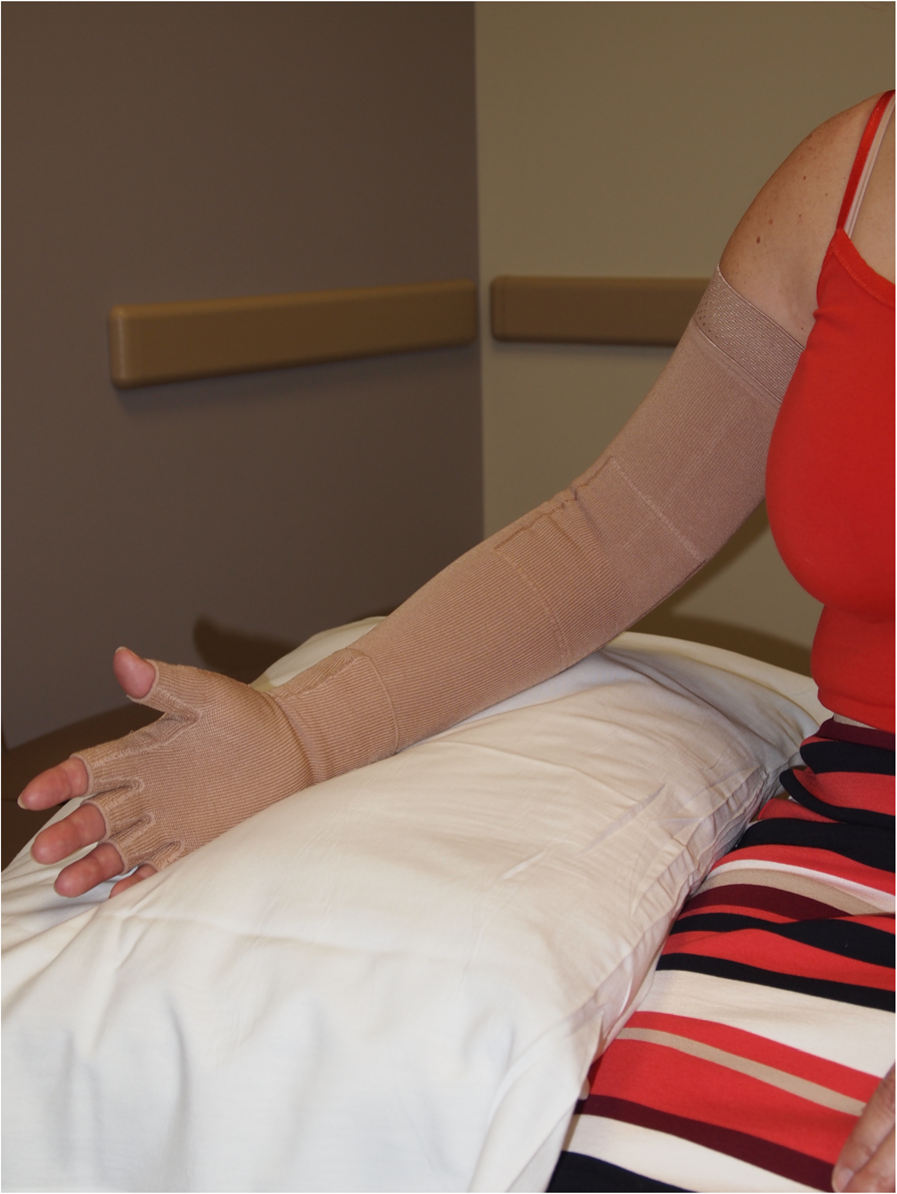
Patient with breast cancer related right upper extremity lymphedema wearing a custom measured and sewn compression garment (sleeve and gauntlet)

High-stretch sports bandages, such as Ace™ wraps, are not recommended for treating lymphedema. Given that a low-stretch bandage has a low resting pressure, the bandage can be worn during the day and at night. During Phase II, it is still recommended that a patient wear the low-stretch bandages at night [48]. Once reduction has been optimized a compression garment, preferably a flat knit garment is recommended and used during the day (Fig. 10). This affords the patient greater freedom and ease of use.

Exercise is an integral part of CDT and should be individualized for the patient based on present level of activity and other co-morbidities. Myths about exercise negatively impacting lymphedema have been dispelled in the literature [15, 41, 55–57].

Lymphedema can predispose a patient to skin breakdown, infection, and delayed wound healing. Meticulous attention to skin care and protection of the edematous limb are essential elements of self-management of lymphedema [58, 59].

#### Lymphedema prevention for at risk limbs

Patients who undergo oncologic treatment are at risk for developing lymphedema when the nodes to be sampled or removed for oncologic reasons also serve to drain the adjacent extremities. It has been strongly recommended for breast cancer patients who are at risk for developing lymphedema because of aforementioned treatments, to avoid intravenous blood draws and intravenous catheter placements in the at risk limb. Additionally, avoidance of blood pressure measurements in the limb as well as use of compression garments during air travel are sometimes recommended [41]. These guidelines are ubiquitous in most centers that treat patients with breast cancer. These practices may be difficult and impractical for patients to adhere to as they must be maintained life-long. They can be a source of anxiety if these guidelines are accidently broken. The evidence to support these guidelines is unsubstantiated and mostly anecdotal. Several recent publications provide insight to the association of these potential risk factors with developing lymphedema. From these studies, it appears that there is very little association with blood draws, injections, or blood pressure measurements in the clinically normal, at-risk limb for developing lymphedema [41, 60]. Additionally, air travel without the use of compression garments was not shown to put patients at risk for lymphedema [61, 62]. These studies could help alleviate some of the anxiety for breast cancer patients who are already under the significant stress burden of having treatment for their malignancy.

### Surgical management

Surgical treatment of lymphedema has been utilized since the early 1900s. One of the early methods of managing lymphedema surgically involved using a silk suture that was threaded in a subcutaneous plane along the affected extremity. This method was believed to bridge the area of lymphatic obstruction and to help establish a lymphatic conduit for egress of edema out of the affected extremity. This was fraught with complications as the material would commonly extrude or become infected [63]. Since that time, numerous procedures have been developed to help manage lymphedema.

Suami and Chang classified the surgical management of lymphedema as either ablative or physiologic. In ablative surgery, the soft tissues, which are edematous and fibrotic, those above the level of the deep fascia, are surgically removed with either direct excision or by liposuction, also termed suction assisted protein lipectomy when performed on a lymphedematous limb [64].

Physiologic methods are those that recreate normal or alternate avenues for lymph fluid to flow out of the affected limb. Two main physiologic interventions are currently employed to treat lymphedema. One is based on the creation of shunts between the congested lymphatic channels and the venous system proximal to the site of lymphatic obstruction. The other relies on the introduction of vascularized soft tissue flaps which frequently include vascularized lymph nodes to the affected extremity [63, 64].

#### Ablative surgical methods

Several ablative procedures have been described, all of which aim at surgical removal of the tissue layers affected by lymphedema, the deep fat compartment above the deep fascia, the superficial fat compartment above the superficial fascia and below the dermis, and to varying degrees the skin itself. The first ablative and most radical method utilized in lower extremity lymphedema was the Charles procedure. In this operation, all of the involved skin and subcutaneous tissue above the investing fascia of the affected extremity is excised. This includes all those tissues just above the muscular compartments of the leg. The resulting wound is then covered with split thickness skin grafts which may be harvested from the affected limb prior to excision of the tissue or from other parts of the body [65]. Sistrunk described a technique of excising wedges of skin and underlying fat down to the level of the deep fascia and closing these incisions primarily. This was most commonly used to reduce thigh circumference [66].

Homan described a modification of the Charles procedure that preserved the overlying skin. This entails a staged procedure where a longitudinal incision is made along the lateral or medial aspect of the leg, lifting the dermis off the underlying fat, while preserving the subdermal plexus. The underlying fat is then excised in the same fashion as described by Charles and the skin flaps are trimmed appropriately to accommodate the limb volume reduction and closed over drains. In a second stage, 3 months later, the procedure is repeated on the previously un-operated side of the same limb [67, 68].

Thompson et al. utilized modifications of this technique for the upper extremity. These modifications that have also been applied to the lower extremities included excising affected tissue, then creating de-epithelialized dermal flaps and folding these in toward, and suturing them to the deep investing fascia, postulating that these dermal bridges would act as connections between deeper lymphatics and vessels and superficial lymphatics facilitating fluid transport [69]. There is no evidence to support that this takes place [64].

Currently, it is felt that these ablative methods should be reserved for more advanced lymphedema that has undergone architectural changes in the soft tissue and would thus be unresponsive to physiologic methods of intervention. This is because once fibrosis and lipodystrophy occur in the affected limb, correcting the fluid accumulation in the limb will not address the fat related volume excess of the extremity that accumulated as a result of the lymphedema or the fibrotic changes that have taken place. In these instances, removing the adipose deposits via liposuction or directly excising the soft tissue is the only intervention that will decrease the size of the limb.

More recently, suction-assisted lipectomy/liposuction has been utilized as an ablative method to remove the hypertrophied fat of the affected extremity. This method of reducing volume of the affected extremity is much less morbid than direct excision procedures. No skin grafting is involved and external scarring at surgical sites is minimal. Some experts feel that liposuction for treatment of lymphedema associated excess fat deposition should be a first line surgical measure for lymphedema not well controlled by nonoperative means.

Because liposuction does not affect the fluid burden attributed to disrupted lymphatics, compression garments or wraps must be worn throughout the day following liposuction to continue to control the excess fluid component and maintain the volume reduction that was achieved [64]. Excellent long term (greater than 15 year follow-up) results have been shown in properly selected patients utilizing liposuction [70].

Patients should still be considered as candidates for a physiologic procedure if pitting edema is present. Currently, most practitioners are looking at combining physiologic procedures and liposuction to address patients who have later stages of lymphedema with significant amounts of fat hypertrophy and/or fibrosis [71].

#### Physiologic surgical methods

Physiologic methods are aimed at reducing the lymphatic fluid burden in lymphedema patients by either improving lymphatic circulation by introducing healthy distant tissue into the affected limb or creating an alternate outflow pathway for lymph fluid. The most common method for creating an alternative outflow is by utilizing the venous circulation, which has the capacity to accommodate the additional fluid load. Most physiologic methods have been used for secondary lymphedema, but some have reported benefit when these procedures are used in primary lymphedema [72].

#### Lymphaticolymphatic bypass

Surgical interventions involving lymphatic vessels directly, were first described by Jackobsen and Suarez in 1962 [73]. Baumeister et al. in the 1990s described the treatment of lymphedema by using healthy lymphatic grafts from the lower extremity as a means of bypassing upper arm lymphatics into healthy neck lymphatics across the scarred axillae of affected upper extremities [74]. Lymphatics in the upper extremity were anastomosed to the lymphatic graft (harvested from a healthy lower extremity) and this graft was then anastomosed to lymphatic vessels in the supraclavicular area. Volume reduction in the extremity was maintained over a period of 3 years [74]. However, this method did have a potential risk of lymphedema developing in the donor extremity. Additionally, a long scar at the donor site and in the affected limb are additional drawbacks of this procedure. Campisi et al. had a similar approach to bypassing the lymphatic obstruction, but instead of using lymphatic vessels as graft conduits, vein grafts from the thigh were used. This helped alleviate potential disruption of the lymphatic system and the risk of additional secondary lymphedema at the donor site [63].

#### Lymphaticovenular anastomosis (LVA)

**Fig. 11:**
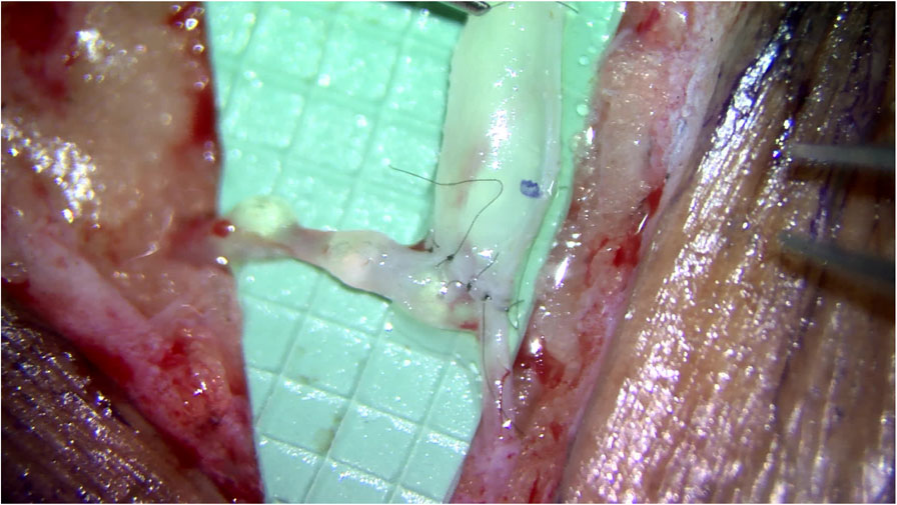
End-to-side anastomosis of 1.5 mm diameter venule into 0.6 mm diameter lymphatic channel in the lower extremity through a 2.5 cm long skin incision

Lymphaticovenular anastomosis (LVA) or Lymphovenous bypass is a surgical procedure where lymphatic vessels in a lymphedematous limb are connected to nearby small veins and venules using microsurgical and super-microsurgical techniques (Fig. 11). This allows the clearance of lymphatic fluid from the affected limb, by utilizing the venous system, which has the capacity to easily accommodate the extra fluid volume, as an outflow. A direct surgical connection is created between the overloaded lymphatic channels, proximal to the site of the lymphatic obstruction, and nearby venules. The concept of this procedure was first introduced in the 1960s [75]. O’Brien reported good results using LVA in the upper extremity as early as 1977 [76]. There has been an increased interest in the use of this surgical method as a treatment modality for lymphedema as imaging techniques (such as intra-operative ICG-directed lymphography), better operating microscopes and microsurgical instrumentation and sutures have become available and have improved the surgeon’s ability to find lymphatic vessels appropriate for LVA and perform supermicrosurgical anastomosis with vessels as small at 0.2 mm in diameter. Numerous studies have been published which show improved symptoms and as much as 61% reduction in volume difference of the affected limb after LVA at one year [31]. A study by Chang et al. showed that in 100 consecutive LVAs, patients who had earlier stage of disease (MD Anderson Stage I/II) and more anastomosis performed had better outcomes. Another finding from this study was that LVAs performed in patients who had lower extremity lymphedema did not have as much improvement as those who had LVAs performed in their upper extremity [31]. One significant benefit of the procedure is that it involves very little surgical site morbidity as the incisions are localized to the affected limb and are 1–2 cm in length. Patients are typically discharged to home the day of surgery or after 23 h of observation in the hospital. In some centers this surgery is performed under local anesthesia.

#### Vascularized lymph node transfer (VLNT)

**Fig. 12:**
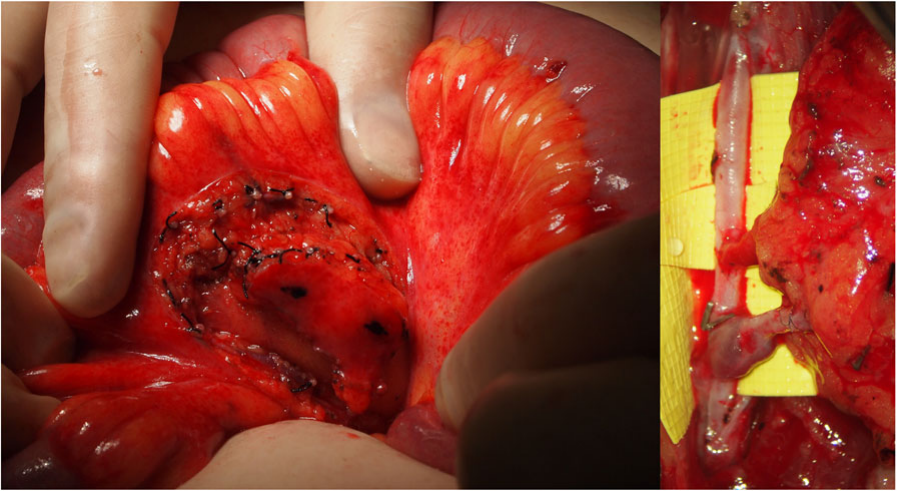
Mesenteric lymph node flap harvested adjacent to the jejunum (*left*) and flap inset with end-to-side anastomoses to the anterior tibial vessels (*right*)

The use of vascularized lymph nodes in the treatment of lymphedema has been evaluated since the 1990s [77]. Transplantation of lymph nodes with a blood supply is termed vascularized lymph node transfer (VLNT). This procedure involves harvesting a lymph node or several lymph nodes along with their vascular supply from a donor site and transferring this vascularized tissue to the affected extremity as a free tissue transfer. A microsurgical anastomosis is performed between the blood vessels of the lymph node flap and the recipient site vessels thus establishing blood flow to the lymph node flap (Fig. 12). Donor sites typically used for lymph node harvest include axillary/lateral thoracic lymph nodes, inguinal lymph nodes, submental lymph nodes, supraclavicular lymph nodes, omental lymph nodes, and mesenteric lymph nodes [64].

There are several theories as to how transferring vascularized lymph nodes to an affected extremity could improve lymphedema. One theory is that lymphatic vessel angiogenesis occurs due to cytokines from the transplanted lymph node flap, establishing lymphatic connections between the lymphatics in the transplanted flap and those of the surrounding lymphatics of the affected limb; specifically VEGF-C has been identified as the growth factor responsible for this lymphangiogenesis. This has been termed a “lymphangiogenetic” mechanism [76]. Another proposed mechanism of action is that the lymph node flap acts as a pump that leads to active removal of lymph fluid from the limb and returning this fluid into the systemic circulation via the flaps venous drainage system [9, 78, 79]. There is experimental support for both of these theories but additional work is needed to further explore the contribution of each. Release of scar tissue and its replacement with a vascularized, non-irradiated soft tissue (the lymph node flap), has also been described as one of the theoretical mechanisms by which the volume reduction may occur. The theory is that any marginally functioning lymphatic channels encased in scar tissue are freed and allowed to drain more effectively with the removal of the scar tissue. Also, the soft tissue flap may act as a bridge, providing lymphatic channels that can re-connect with channels in the affected limb distally and proximally to the channels unaffected by surgery and radiation therapy, thereby providing a type of interposition lymphatic graft that will facilitate fluid transport across the previously treated, scarred areas [80].

An additional postulated positive effect is the introduction of the lymph node flap as an immune system organ. Lymphatic channels from the affected limb connect with the lymphatics of the lymph node flap and present antigens to the lymph nodes which can than mount an immune response and minimize the risk of infection for the lymphedematous limb. This has been indirectly observed in that most patients with recurrent bouts of cellulitis in the lymphedematous limb pre-operatively, will experience a significant reduction of these episodes after the VLNT [9, 79].

To date, several studies have shown that vascularized lymph node transfer is useful in reducing lymphedema. One of the larger studies by Becker et al. evaluated 1500 patients with stage I, II, and III lymphedema who had undergone vascularized lymph node transfer. The minimum follow up was 3 years. Findings included a 98% subjective improvement. Forty percent of patients with stage I and stage II lymphedema had significant improvement and required no further conservative therapy. For patients with stage III lymphedema, 95% had some improvement and 98% remained infection free. However, the stage III patients still required conservative therapy to help control edema in the limb [80].

One of the major drawbacks to lymph node transfers is the potential for iatrogenic secondary lymphedema at the donor sites. Secondary lymphedema in the ipsilateral lower extremity after groin lymph node harvest and the ipsilateral upper extremity after axillary/lateral thoracic, level 5 neck nodes, and supraclavicular lymph node harvest have all been reported. There are no quoted percentages for the rate of lymphedema occurrence from donor sites, however; when discussing outcomes with leaders in the field of surgical lymphedema treatment, it is a universally feared complication. One method, developed to help prevent this complication is to use reverse mapping to identify and protect lymph nodes that preferentially drain the extremity and to spare these during a groin lymph node or axillary/lateral thoracic lymph node harvest. This is done by injecting radiotracer (Technetium-99 m sulfur colloid) into the subdermal plane at the web spaces of the ipsilateral limb and ICG into the subdermal plane of the ipsilateral groin or axilla. Intraoperatively the lymph nodes identified by the ICG dye are also examined with a gamma probe. Any “hot” lymph nodes, that elicit radiotracer uptake are spared and not included in the flap harvest, thus, in theory, preserving the lymph nodes, which preferentially drain the donor extremity. Dayan et al. have published very promising preliminary results using this method [34].

#### Vascularized omental flap transfer

**Fig. 13:**
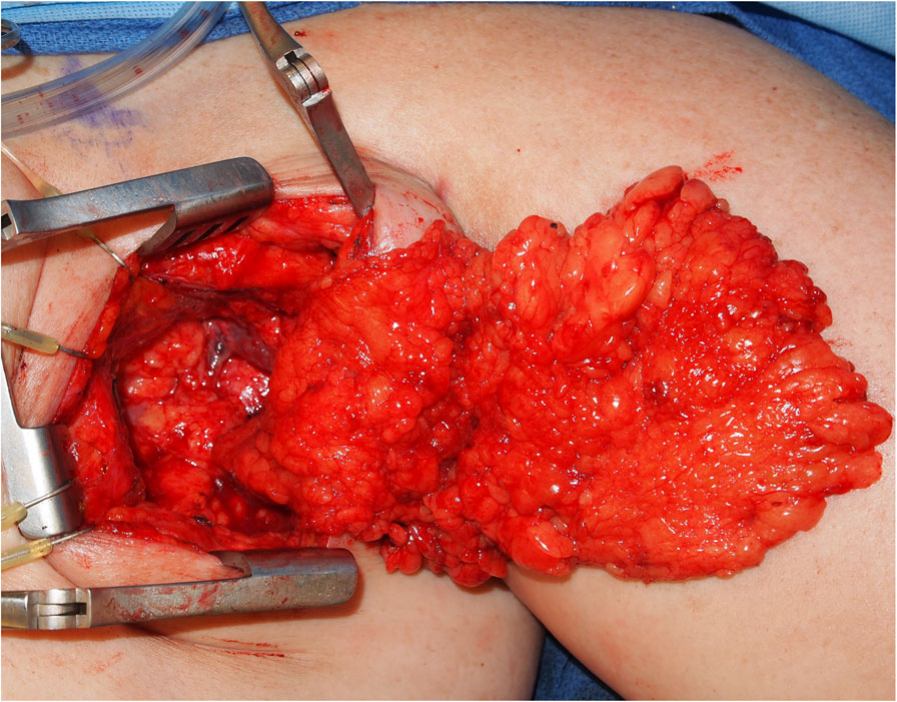
Segmental omental flap to the left axilla after extensive scar excision for breast cancer associated left upper extremity lymphedema

Because of the risk of donor site lymphedema, clinicians have sought out other sources of vascularized lymphatic tissue. The omentum’s function as lymphatic organ has been explored for possible applications in lymphedema management. Surgeons began using the omentum as a pedicled flap attached to its gastroepiploic vascular supply to aid with lymphatic drainage in upper extremity lymphedema beginning in the 1960s. This involved significant morbidity as a full celiotomy was performed and the flap had to be tunneled to the recipient site as free tissue transfer had not yet been discovered. Since that time, use of the omentum in the treatment of lymphedema has been revisited as a free tissue transfer (Fig. 13). To help minimize morbidity from the harvest site, minimally invasive approaches for harvest, using laparoscopy, have been used and are garnering more interest. Results from this approach have yet to be fully validated, but early reports show promising results similar to vascularized lymph node transfers from other sites [81].

Similarly, our group at The Ohio State University has recently described the use of mesenteric lymph nodes as free flaps for lymphedema with early promising results and minimal donor site morbidity [82].

#### Simultaneous microsurgery breast reconstruction and vascularized lymph node transfer

Recently, the use of a combined abdominal and groin lymph node flap has been utilized to treat lymphedema in the upper extremity for patients who have undergone mastectomy and axillary lymph node sampling and have developed refractory lymphedema. Results from this approach are limited due to the lack of data currently available, but some improvement has been noted. This approach involves the harvest of a cluster of nodes surrounding the superficial inferior epigastric (SIE) vessels on the side contralateral to the deep inferior epigastric vessels used to revascularize the abdominal tissue, used for the breast mound reconstruction, to the internal mammary vessels. The SIE vessels are anastomosed to vessel in the axilla to augment the blood supply and, perhaps even more importantly the venous drainage of the lymph nodes that are placed toward the axilla [83]. The challenges with this operation is the appropriate placement of the lymph nodes into the axilla to allow the upper extremity lymphatics to connect to the lymphatics of the transferred nodes. Chang and Nguyen et al. presented a summary of geometric arrangements for various breast reconstruction scenarios. The vascularity of this lymph node cluster may be compromised as the harvest proceeds relatively blindly around the nodes and the surrounding fat tissue down to the SIE vessel take off from the femoral vessels, not allowing visualization of the nodal blood supply itself. There is also a theoretical risk for lymphedema to the ipsilateral lower extremity with this lymph node flap harvest [83].

#### Comparison of surgical procedures

One of the problems in monitoring success of surgical interventions is that there is no set standard for measuring degree of lymphedema and no standardized conservative treatment protocol before or after surgery. There is currently no uniformity in the literature with regards to a protocol for diagnosing and monitoring lymphedema. Clinicians who follow these patients have reported objective and subjective improvements in the majority of lymphedema patients who have undergone surgical intervention. Most studies that report on the surgical management of lymphedema monitor limb circumference, volume reduction, and incidence of cellulitis as their endpoints. More recently, patient self-reported quality of life outcome tools specific for lymphedema have been included as an additional meaningful end point.

The most commonly performed surgical procedures for lymphedema are LVA and vascularized lymph node transfer. Several meta-analysis have looked at the safety and efficacy of the surgical procedures commonly performed for lymphedema and have found that there is evidence that patients who undergo LVA or vascularized lymph node transfer have quantitative improvement of lymphedema [78, 82, 84]. Additionally, more than 90% of patients in the studies included in the meta-analysis reported an improvement subjectively. Patients who had vascularized lymph node transfer had a slightly better improvement in symptoms, but there is no current evidence available to strongly support one procedure over the other. Surgeons who perform these procedures often choose their operative approach on a case-by-case basis using clinical judgment as a guide. LVA is generally offered first if it is deemed feasible because it is a less invasive procedure and there is no risk of a donor site as seen with vascularized lymph node transfers. In select patients, a combination of both procedures may be efficacious. Messia et al. have developed an algorithm that includes both treatment modalities in the treatment of breast cancer related lymphedema. The Barcelona Lymphedema Algorithm for Surgical Treatment of Lymphedema goes through a series of steps which evaluated the functionality of the lymphatics of the affected limb. For patients who have both functioning lymphatics and significant scarring in the axilla, after scar release, autologous vascularized lymph nodes are transferred into the axilla, and during this same procedure, LVA(s) are performed. This approach has shown good preliminary results [85, 86].

#### Prophylactic surgical measures to prevent lymphedema

One of the methods currently being employed to prevent lymphedema from occurring after axillary lymph node samplings is termed Lymphatic Microsurgical Preventative Healing Approach (LYMPHA). This method, championed in Italy, utilizes lymphovenous anastomoses of upper extremity lymphatics at the time of the axillary node dissection to bypass any severed arm lymphatics immediately. Patients with BMI greater than 30 or those with normal BMI and impaired lymphatic function as seen during lymphoscintigraphy are selected to undergo the prophylactic LVA procedure immediately after lymph node sampling. The LVA was performed by identifying divided upper extremity lymphatics in the axilla and performing an end to side anastomosis to a branch of the axillary vein or an end to end anastomosis to one of its side branches. Of the 74 patients who were treated with the LYMPHA approach, 3 developed lymphedema (4%). This number is lower than the incidence of lymphedema reported after sentinel lymph node biopsy and full lymph node dissection (6–13% and 13–65%, respectively). As these patients all underwent an axillary node dissection, this represented an order of magnitude reduction in the incidence of lymphedema. Patients who remained lymphedema free were followed out to 4 years. This approach should be considered for all at risk patients (those undergoing axillary node dissection, especially in the obese patient) [87].

## Current research and the future of secondary lymphedema management

### Genetics related to secondary lymphedema

In an effort to identify and intervene early in the management of lymphedema, researchers are currently investigating additional factors, beyond node dissection, radiation therapy to nodal basins, chemotherapy, and obesity that may put patients at risk for developing lymphedema. With early intervention, preventive measures, including surgery, could be utilized for those at highest risk for development of the disease. One current idea is that genetics may put some patients at higher risk for developing secondary lymphedema. Primary lymphedema is known to have a genetic etiology, but a group of researchers at the University of Pittsburgh have been looking at a possible genetic component in secondary lymphedema. Their case-control evaluation of 188 women diagnosed with breast cancer from 2000 to 2010 has shown that a gene mutation of the gene connexin-47 was identified in a greater number of women who developed secondary lymphedema. Connexin-47 is thought to be important for the development of valves within normal lymphatics and its absence may result in lymphatic dysfunction. This study has challenged the notion that secondary lymphedema in cancer patients is due to treatment effects alone causing the malfunction of lymphatic transport, but rather suggests that predisposing genetic factors may play an important role also [88]. More research is needed to elucidate the mechanism by which this may be a contributing factor to the development of secondary lymphedema in humans.

### Timing of intervention

Another currently debated topic related to lymphedema surgery involves timing of interventions. Current practices dictate that conservative measure be utilized first when patients are initially diagnosed with lymphedema. This paradigm is essentially ubiquitous in western medicine. Conservative methods are tried first and if these fail, more invasive methods, such as surgery, are considered. This mantra, however, seems to not be necessarily true for the treatment in lymphedema. Some feel that a certain window may be missed if surgery is considered a “last ditch” intervention. This is especially true with the physiologic procedures that rely on a residual functioning lymphatic system in the affected limb to be most successful. It is thought that by allowing the disease to progress to later stages, the remaining lymphatics may be so injured from being overwhelmed by the excess lymphatic fluid build-up, which will cause localized fibrosis, that they may no longer be suitable for bypass procedures [64]. This is evident by ICG lymphangiograms that show no superficial lymphatic channels available for bypass in later stages of lymphedema. Because of this, most surgeons recommend physiologic procedures for patients that have stage II or early stage III lymphedema. Furthermore, some believe that Stage I lymphedema patients and patients who are at high risk for lymphedema would benefit from having early or prophylactic physiologic procedures, respectively. In a study by Becker et al., results from vascularized lymph node transfer suggested that patients who had surgical interventions earlier in the course of their disease had better outcomes, more complete resolution of their edema in a greater number of patients [89].

### Role of cytokines

Some researchers are looking at ways that can promote regeneration of lymphatics in areas where they have been injured and lymphedema has developed. Vascular Endothelial Growth Factor-C (VEGF-C) has been found to be involved in lymphangiogenesis and the development of the lymphatic system. Defects in genes that produce VEGF-C are the underlying cause to some forms of primary lymphedema. By using isolated VEGF-C in these injured areas, one research group was able to show better regeneration of lymphatics when used in combination with lymph node transfers in the animal model. Current human trials are underway [90]. VEGF-C is also being investigated in conjunction with bioengineered scaffolds to regenerate damaged or missing lymphatic channels.

### Future direction of lymphatic surgery

The future direction of lymphatic surgery will allow for the design of personalized surgical management strategies that best address the needs of the individual cancer patient. In this regard, there are several different methodological and management aspects of lymphatic surgery that are actively being further pursued at this time. This includes: (i) advancements in real-time intraoperative lymphatic mapping techniques; (ii) the development and use of bioengineered lymphatic conduits; (iii) the refinement of “dual-level” VLNT techniques (i.e., such as simultaneous VLNT in both the proximal/axilla region and distal/wrist region); (iv) combined VLNT and LVA techniques; (v) expansion of the role of prophylactic LVA; and (vi) combining pharmacologic therapies with physiologic surgical methods. The success of these cutting-edge advancements will obviously help to shape the future direction of lymphatic surgery, thus leading to more complete and sustainable treatment strategies.

## Conclusions

Over the last 100 years, we have learned a significant amount about the lymphatic system and, more recently, we have begun to understand the pathogenesis and treatment of lymphedema. However, the challenges to successfully managing lymphedema remain formidable. Clinical research efforts are beginning to show promising results that could ultimately lead to more complete and sustainable treatment strategies and perhaps a cure for secondary lymphedema and its devastating resultant morbidities.

## Abbreviations

BMI: Body mass index
CDT: Complex decongestive therapy or complete decongestive therapy
ICG: Indocyanine green
LVA: Lymphaticovenular anastomosis
MLD: Manual lymphatic drainage
SIE: Superficial inferior epigastric
VD: Volume differential
VDR: Volume differential reduction
VLNT: Vascularized lymph node transfer
WAC: Weight-adjusted volume

## References

[1] PDQ Supportive and Palliative Care Editorial Board (2002). Lymphedema (PDQ®): Health Professional Version. 2015 Jul 17. PDQ Cancer Information Summaries [Internet].

[2] Warren AG, Brorson H, Borud LJ, Slavin SA (2007). Lymphedema: a comprehensive review. Ann Plast Surg.

[3] Szuba A, Shin WS, Strauss HW, Rockson S (2003). The third circulation: radionuclide lymphoscintigraphy in the evaluation of lymphedema. J Nucl Med.

[4] Fu MR, Ridner SH, Hu SH, Stewart BR, Cormier JN, Armer JM (2013). Psychosocial impact of lymphedema: a systematic review of literature from 2004 to 2011. Psychooncology.

[5] De Brucker B, Zeltzer A, Seidenstuecker K, Hendrickx B, Adriaenssens N, Hamdi M (2016). Breast cancer-related lymphedema: quality of life after lymph node transfer. Plast Reconstr Surg.

[6] Mortimer PS, Rockson SG (2014). New developments in clinical aspects of lymphatic disease. J Clin Invest.

[7] Petrova TV, Karpanen T, Norrmén C, Mellor R, Tamakoshi T, Finegold D, Ferrell R, Kerjaschki D, Mortimer P, Ylä-Herttuala S, Miura N, Alitalo K. Defective valves and abnormal mural cell recruitment underlie lymphatic vascular failure in lymphedema distichiasis. Nat Med. 2004;10(9):974–81. 10.1038/nm1094 15322537

[8] Mescher A, Junqueira LC (2013). Junqueira's Basic Histology: Text and Atlas.

[9] Cheng MH, Huang JJ, Wu CW, Yang CY, Lin CY, Henry SL, Kolios L. The mechanism of vascularized lymph node transfer for lymphedema: natural lymphaticovenous drainage. Plast Reconstr Surg. 2014;133(2):192e–8e. 10.1097/01.prs.0000437257.78327.5b 24469190

[10] Murdaca G, Cagnati P, Gulli R, Spanò F, Puppo F, Campisi C, Boccardo F. Current views on diagnostic approach and treatment of lymphedema. Am J Med. 2012;125(2):134–40. 10.1016/j.amjmed.2011.06.032 22269614

[11] Hinrichs CS, Gibbs JF, Driscoll D, Kepner JL, Wilkinson NW, Edge SB, Fassl KA, Muir R, Kraybill WG. The effectiveness of complete decongestive physiotherapy for the treatment of lymphedema following groin dissection for melanoma. J Surg Oncol. 2004;85(4):187–92. 10.1002/jso.20020 14991874

[12] Chance-Hetzler J, Armer J, Van Loo M, Anderson B, Harris R, Ewing R, Stewart B. Prospective Lymphedema Surveillance in a Clinic Setting. J Pers Med. 2015;5(3):311–25. 10.3390/jpm5030311 PMC4600150 26308061

[13] Kim M, Kim SW, Lee SU, Lee NK, Jung SY, Kim TH, Lee ES, Kang HS, Shin KH. A model to estimate the risk of breast cancer-related lymphedema: combinations of treatment-related factors of the number of dissected axillary nodes, adjuvant chemotherapy, and radiation therapy. Int J Radiat Oncol Biol Phys. 2013;86(3):498–503. 10.1016/j.ijrobp.2013.02.018 23541809

[14] Liljegren G, Holmberg L (1997). Arm morbidity after sector resection and axillary dissection with or without postoperative radiotherapy in breast cancer stage I. Results from a randomised trial. Uppsala-Orebro Breast Cancer Study Group. Eur J Cancer.

[15] Sagen A, Kåresen R, Risberg MA (2009). Physical activity for the affected limb and arm lymphedema after breast cancer surgery. A prospective, randomized controlled trial with two years follow-up. Acta Oncol.

[16] Akita S, Mitsukawa N, Rikihisa N, Kubota Y, Omori N, Mitsuhashi A, Tate S, Shozu M, Satoh K. Early diagnosis and risk factors for lymphedema following lymph node dissection for gynecologic cancer. Plast Reconstr Surg. 2013;131(2):283–90. 10.1097/PRS.0b013e318277870f 23357989

[17] Fu MR (2014). Breast cancer-related lymphedema: symptoms, diagnosis, risk reduction, and management. World J Clin Oncol.

[18] Cormier JN, Xing Y, Zaniletti I, Askew RL, Stewart BR, Armer JM (2009). Minimal limb volume change has a significant impact on breast cancer survivors. Lymphology.

[19] Czerniec SA, Ward LC, Refshauge KM, Beith J, Lee MJ, York S, Kilbreath SL. Assessment of breast cancer-related arm lymphedema--comparison of physical measurement methods and self-report. Cancer Invest. 2010;28(1):54–62. 10.3109/07357900902918494 19916749

[20] Armer JM, Stewart BR (2005). A comparison of four diagnostic criteria for lymphedema in a post-breast cancer population. Lymphat Res Biol.

[21] Maclellan RA, Couto RA, Sullivan JE, Grant FD, Slavin SA, Greene AK (2015). Management of primary and secondary lymphedema: analysis of 225 referrals to a center. Ann Plast Surg.

[22] Brinegar KN, Sheth RA, Khademhosseini A, Bautista J, Oklu R (2015). Iliac vein compression syndrome: clinical, imaging and pathologic findings. World J Radiol.

[23] Suliman A, Greenberg JI, Angle N (2008). Surgical bypass of symptomatic central venous obstruction for arteriovenous fistula salvage in hemodialysis patients. Ann Vasc Surg.

[24] Data presented at 41ST EUROPEAN SOCIETY OF LYMPHOLOGY (ESL) CONGRESS, June 4–6, 2015 by Jean-Paul Belgrade (PhD) and Lisbeth Vandermeeren (MD).

[25] DiSipio T, Rye S, Newman B, Hayes S (2013). Incidence of unilateral arm lymphoedema after breast cancer: a systematic review and meta-analysis. Lancet Oncol.

[26] Williams A (2015). Measuring change in limb volume to evaluate lymphoedema treatment outcome. EWMA J.

[27] Beek MA, te Slaa A, van der Laan L, Mulder PG, Rutten HJ, Voogd AC, Luiten EJ, Gobardhan PD. Reliability of the Inverse Water Volumetry Method to Measure the Volume of the Upper Limb. Lymphat Res Biol. 2015;13(2):126–30. 10.1089/lrb.2015.0011 26091408

[28] International Society of Lymphology (ISL) (2013). The diagnosis and treatment of peripheral lymphoedema. Consensus document of the International Society of Lymphology. Lymphology.

[29] Chen YW, Tsai HJ, Hung HC, Tsauo JY. Reliability study of measurements for lymphedema in breast cancer patients. Am J Phys Med Rehabil. 2008;87(1):33–8. 10.1097/PHM.0b013e31815b6199 17993983

[30] Ohberg F, Zachrisson A, Holmner-Rocklöv Å. Three-dimensional camera system for measuring arm volume in women with lymphedema following breast cancer treatment. Lymphat Res Biol. 2014;12(4):267–74. 10.1089/lrb.2014.0026 25495382

[31] Chang DW, Suami H, Skoracki R. A prospective analysis of 100 consecutive lymphovenous bypass cases for treatment of extremity lymphedema. Plast Reconstr Surg. 2013;132(5):1305–14. 10.1097/PRS.0b013e3182a4d626 24165613

[32] Ridner SH, Shih YC, Doersam JK, Rhoten BA, Schultze BS, Dietrich MS. A pilot randomized trial evaluating lymphedema self-measurement with bioelectrical impedance, self-care adherence, and health outcomes. Lymphat Res Biol. 2014;12(4):258–66. 10.1089/lrb.2014.0017 PMC4267706 25412401

[33] Borri M, Schmidt MA, Gordon KD, Wallace TA, Hughes JC, Scurr ED, Koh DM, Leach MO, Mortimer PS. Quantitative Contrast-Enhanced Magnetic Resonance Lymphangiography of the Upper Limbs in Breast Cancer Related Lymphedema: An Exploratory Study. Lymphat Res Biol. 2015;13(2):100–6. 10.1089/lrb.2014.0039 PMC4492592 25774851

[34] Dayan JH, Dayan E, Smith ML. Reverse lymphatic mapping: a new technique for maximizing safety in vascularized lymph node transfer. Plast Reconstr Surg. 2015;135(1):277–85. 10.1097/PRS.0000000000000822 25285683

[35] Yamamoto T, Narushima M, Doi K, Oshima A, Ogata F, Mihara M, Koshima I, Mundinger GS. Characteristic indocyanine green lymphography findings in lower extremity lymphedema: the generation of a novel lymphedema severity staging system using dermal backflow patterns. Plast Reconstr Surg. 2011;127(5):1979–86. 10.1097/PRS.0b013e31820cf5df 21532424

[36] Yamamoto T, Yamamoto N, Doi K, Oshima A, Yoshimatsu H, Todokoro T, Ogata F, Mihara M, Narushima M, Iida T, Koshima I. Indocyanine green-enhanced lymphography for upper extremity lymphedema: a novel severity staging system using dermal backflow patterns. Plast Reconstr Surg. 2011;128(4):941–7. 10.1097/PRS.0b013e3182268cd9 21681123

[37] Chang DW, Masia J, Garza R, Skoracki R, Neligan PC (2016). Lymphedema: surgical and medical therapy. Plast Reconstr Surg.

[38] Campisi C, Boccardo F (2004). Microsurgical techniques for lymphedema treatment: derivative lymphatic-venous microsurgery. World J Surg.

[39] Miller CL, Specht MC, Horick N, Skolny MN, Jammallo LS, O'Toole J, Taghian AG. A novel, validated method to quantify breast cancer-related lymphedema (BCRL) following bilateral breast surgery. Lymphology. 2013;46(2):64–74. 24354105

[40] Keeley V, Crooks S, Locke J, Veigas D, Riches K, Hilliam R. A quality of life measure for limb lymphoedema (LYMQOL). Journal of Lymphoedema. 2010;5(1):26–37.

[41] Cemal Y, Pusic A, Mehrara BJ (2011). Preventative measures for lymphedema: separating fact from fiction. J Am Coll Surg.

[42] Moseley AL, Carati CJ, Piller NB (2007). A systematic review of common conservative therapies for arm lymphoedema secondary to breast cancer treatment. Ann Oncol.

[43] Szuba A, Rockson SG (1998). Lymphedema: classification, diagnosis and therapy. Vasc Med.

[44] Kligman L, Wong RK, Johnston M, Laetsch NS (2004). The treatment of lymphedema related to breast cancer: a systematic review and evidence summary. Support Care Cancer.

[45] Boris M (1994). Lymphedema reduction by noninvasive complex lymphedema therapy. Oncology.

[46] Casley-Smith JR (1991). Exercises for Patients with Lymphedema of the Arm, ed. 2.

[47] Holtgrefe KM (2006). Twice-weekly completed decongestive physical therapy in the management of secondary lymphedema of the lower extremities. Phys Ther.

[48] Burt J, White G (1999). Lymphedema: A Breast Cancer Patient’s Guide to Prevention and Healing.

[49] Lawenda BD, Mondry TE, Johnstone PA (2009). Lymphedema: a primer on the identification and management of a chronic condition in oncologic treatment. CA Cancer J Clin.

[50] Boris M, Weindorf S, Lasinski B (1997). Persistence of lymphedema reduction after noninvasive complex lymphedema therapy. Oncology.

[51] Hwang JH, Choi JY, Lee JY, Hyun SH, Choi Y, Choe YS, Lee KH, Kim BT. Lymphoscintigraphy predicts response to complex decongestive therapy in patients with early stage extremity lymphedema. Lymphology. 2007;40:172–6. 18365531

[52] Tan IC, Maus EA, Rasmussen LC, Marshall MV, Adams KE, Fife CE, Smith LA, Chan W, Sevick-Muraca EM. Assessment of lymphatic contractile function following manual lymphatic drainage using near-infrared fluorescence imaging. Arch Phys Med Rehabil. 2011;92(5):756–64. 10.1016/j.apmr.2010.12.027 PMC3109491 21530723

[53] Földi M, Földi E, Kubik S (2003). Textbook of Lymphology.

[54] Zuther JE, Norton S (2013). Lymphedema management: a comprehensive guide for practitioners.

[55] Ammitzbøll G, Lanng C, Kroman N, Zerahn B, Hyldegaard O, Kaae Andersen K, Johansen C, Dalton SO. Progressive strength training to prevent LYmphoedema in the first year after breast CAncer - the LYCA feasibility study. Acta Oncol. 2017;56(2):360–6. 10.1080/0284186X.2016.1268266 28084150

[56] Ahmed RL, Thomas W, Yee D, Schmitz KH. Randomized controlled trial of weight training and lymphedema in breast cancer survivors. J Clin Oncol. 2006;24(18):2765–72. 10.1200/JCO.2005.03.6749 16702582

[57] Courneya KS, Segal RJ, Mackey JR, Gelmon K, Reid RD, Friedenreich CM, Ladha AB, Proulx C, Vallance JK, Lane K, Yasui Y, McKenzie DC. Effects of aerobic and resistance exercise in breast cancer patients receiving adjuvant chemotherapy: a multicenter randomized controlled trial. J Clin Oncol. 2007;25(28):4396–404. 10.1200/JCO.2006.08.2024 17785708

[58] Brennan MJ, DePompodo RW, Garden FH (1996). Focused review: postmastectomy lymphedema. Arch Phys Med Rehabil.

[59] Megens A, Harris S (1998). Physical therapist management of lymphedema following treatment for breast cancer: a critical review of its effectiveness. Phys Ther.

[60] Winge C, Mattiasson AC, Schultz I (2010). After axillary surgery for breast cancer–is it safe to take blood samples or give intravenous infusions?. J Clin Nurs.

[61] Graham PH. Compression prophylaxis may increase the potential for flight-associated lymphoedema after breast cancer treatment. Breast. 2002;11(1):66–71. 10.1054/brst.2001.0370 14965648

[62] Kilbreath SL, Ward LC, Lane K, McNeely M, Dylke ES, Refshauge KM, McKenzie D, Lee MJ, Peddle C, Battersby KJ. Effect of air travel on lymphedema risk in women with history of breast cancer. Breast Cancer Res Treat. 2010;120(3):649–54. 10.1007/s10549-010-0793-3 20180016

[63] Campisi C, Boccardo F (2002). Lymphedema and microsurgery. Microsurgery.

[64] Suami H, Chang DW (2010). Overview of surgical treatments for breast cancer-related lymphedema. Plast Reconstr Surg.

[65] Charles RH (1912). Elephantiasis Scroti.

[66] Sistrunk WE (1927). Contribution to plastic surgery: removal of scars by stages; an open operation for extensive laceration of the anal sphincter; the Kondoleon operation for elephan- tiasis. Ann Surg.

[67] Homans J (1936). The treatment of elephantiasis of the legs. A preliminary report. N Engl J Med.

[68] Miller TA, Wyatt LE, Rudkin GH (1998). Staged skin and subcutaneous excision for lymphedema: a favorable report of long-term results. Plast Reconstr Surg.

[69] Thompson N (1970). Buried dermal flap operation for chronic lymphedema of the extremities. Ten-year survey of results in 79 cases. Plast Reconstr Surg.

[70] Brorson H (2016). Liposuction in lymphedema treatment. J Reconstr Microsurg.

[71] Nicoli F, Constantinides J, Ciudad P, Sapountzis S, Kiranantawat K, Lazzeri D, Lim SY, Nicoli M, Chen PY, Yeo MS, Chilgar RM, Chen HC. Free lymph node flap transfer and laser-assisted liposuction: a combined technique for the treatment of moderate upper limb lymphedema. Lasers Med Sci. 2015;30(4):1377–85. 10.1007/s10103-015-1736-3 25820369

[72] Hara H, Mihara M, Ohtsu H, Narushima M, Iida T, Koshima I (2015). Indication of lymphaticovenous anastomosis for lower limb primary lymphedema. Plast Reconstr Surg.

[73] Jacobson JH, Suarez EL (1962). Microvascular surgery. Dis Chest.

[74] Baumeister RG, Siuda S (1990). Treatment of lymphedemas by microsurgical lymphatic grafting: what is proved?. Plast Reconstr Surg.

[75] Laine JB, Howard JM. Experimental lymphatico-venous anastomosis. Surg Forum. 1963;14:111–2. 14064471

[76] O'Brien BM, Sykes P, Threlfall GN, Browning FS. Microlymphaticovenous anastomoses for obstructive lymphedema. Plast Reconstr Surg. 1977;60(2):197–211. 10.1097/00006534-197708000-00006 887661

[77] Chen HC, O'Brien BM, Rogers IW, Pribaz JJ, Eaton CJ. Lymph node transfer for the treatment of obstructive lymphoedema in the canine model. Br J Plast Surg. 1990;43(5):578–86. 10.1016/0007-1226(90)90123-h 2224354

[78] Raju A, Chang DW (2015). Vascularized lymph node transfer for treatment of lymphedema: a comprehensive literature review. Ann Surg.

[79] Cheng MH, Chen SC, Henry SL, Tan BK, Lin MC, Huang JJ. Vascularized groin lymph node flap transfer for postmastectomy upper limb lymphedema: flap anatomy, recipient sites, and outcomes. Plast Reconstr Surg. 2013;131(6):1286–98. 10.1097/PRS.0b013e31828bd3b3 23714790

[80] Becker C, Vasile JV, Levine JL, Batista BN, Studinger RM, Chen CM, Riquet M. Microlymphatic surgery for the treatment of iatrogenic lymphedema. Clin Plast Surg. 2012;39(4):385–98. 10.1016/j.cps.2012.08.002 23036289

[81] Nguyen AT, Suami H (2015). Laparoscopic free omental lymphatic flap for the treatment of lymphedema. Plast Reconstr Surg.

[82] Coriddi M, Skoracki R, Eiferman D. Vascularized jejunal mesenteric lymph node transfer for treatment of extremity lymphedema. Microsurgery. 2017;37(2):177–8. 10.1002/micr.30037 26892278

[83] Nguyen AT, Chang EI, Suami H, Chang DW (2015). An algorithmic approach to simultaneous vascularized lymph node transfer with microvascular breast reconstruction. Ann Surg Oncol.

[84] Basta MN, Gao LL, Wu LC (2014). Operative treatment of peripheral lymphedema: a systematic meta-analysis of the efficacy and safety of lymphovenous microsurgery and tissue transplantation. Plast Reconstr Surg.

[85] Masia J, Pons G, Nardulli ML. Combined Surgical Treatment in Breast Cancer-Related Lymphedema. J Reconstr Microsurg. 2016;32(1):16–27. 10.1055/s-0035-1544182 25868153

[86] Masià J, Pons G, Rodríguez-Bauzà E. Barcelona Lymphedema Algorithm for Surgical Treatment in Breast Cancer-Related Lymphedema. J Reconstr Microsurg. 2016;32(5):329–35. 10.1055/s-0036-1578814 26975564

[87] Boccardo F, Casabona F, De Cian F, Friedman D, Murelli F, Puglisi M, Campisi CC, Molinari L, Spinaci S, Dessalvi S, Campisi C. Lymphatic microsurgical preventing healing approach (LYMPHA) for primary surgical prevention of breast cancer-related lymphedema: over 4 years follow-up. Microsurgery. 2014;34(6):421–4. 10.1002/micr.22254 24677148

[88] Finegold DN, Baty CJ, Knickelbein KZ, Perschke S, Noon SE, Campbell D, Karlsson JM, Huang D, Kimak MA, Lawrence EC, Feingold E, Meriney SD, Brufsky AM, Ferrell RE. Connexin 47 mutations increase risk for secondary lymphedema following breast cancer treatment. Clin Cancer Res. 2012;18(8):2382–90. 10.1158/1078-0432.CCR-11-2303 PMC3625665 22351697

[89] Becker C, Assouad J, Riquet M, Hidden G (2006). Postmastectomy lymphedema: long-term results following microsurgical lymph node transplantation. Ann Surg.

[90] Schindewolffs L, Breves G, Buettner M, Hadamitzky C, Pabst R (2014). VEGF-C improves regeneration and lymphatic reconnection of transplanted autologous lymph node fragments: an animal model for secondary lymphedema treatment. Immun Inflamm Dis.

